# A comparison of BMP2 delivery by coacervate and gene therapy for promoting human muscle-derived stem cell-mediated articular cartilage repair

**DOI:** 10.1186/s13287-019-1434-3

**Published:** 2019-11-26

**Authors:** Xueqin Gao, Haizi Cheng, Hassan Awada, Ying Tang, Sarah Amra, Aiping Lu, Xuying Sun, Guijin Lv, Charles Huard, Bing Wang, Xiaohong Bi, Yadong Wang, Johnny Huard

**Affiliations:** 1 Department of Orthopaedic Surgery, University of Texas Health Science Center at Houston, Houston, TX USA; 2 Institute of Molecular Medicine, McGovern Medical School, University of Texas Health Science Center at Houston, Houston, TX USA; 3 Department of Center for Regenerative Sports Medicine, Steadman Philippon Research Institute, Vail, CO USA; 4 Meinig School of Biomedical Engineering, Cornell University, Ithaca, NY USA; 5 Department of Orthopaedic Surgery, University of Pittsburgh, Pittsburgh, PA USA; 6 Department of Nanomedicine, Institute of Molecular Medicine, McGovern Medical School, University of Texas Health Science Center at Houston, Houston, TX USA

**Keywords:** Bone morphogenetic proteins 2, Human muscle-derived stem cells, Coacervate, Osteoarthritis, Soluble fms-like tyrosine kinase-1, Cartilage repair

## Abstract

**Background:**

Osteoarthritis and cartilage injury treatment is an unmet clinical need. Therefore, development of new approaches to treat these diseases is critically needed. Previous work in our laboratory has shown that murine muscle-derived stem cells (MDSCs) can efficiently repair articular cartilage in an osteochondral and osteoarthritis model. However, the cartilage repair capacity of human muscle-derived stem cells has not been studied which prompt this study.

**Method:**

In this study, we tested the in vitro chondrogenesis ability of six populations of human muscle-derived stem cells (hMDSCs), before and after lenti-BMP2/GFP transduction using pellet culture and evaluated chondrogenic differentiation of via histology and Raman spectroscopy. We further compared the in vivo articular cartilage repair of hMDSCs stimulated with BMP2 delivered through coacervate sustain release technology and lenti-viral gene therapy-mediated gene delivery in a monoiodoacetate (MIA)-induced osteoarthritis (OA) model. We used microCT and histology to evaluate the cartilage repair.

**Results:**

We observed that all hMDSCs were able to undergo chondrogenic differentiation in vitro. As expected, lenti-BMP2/GFP transduction further enhanced the chondrogenic differentiation capacities of hMDSCs, as confirmed by Alcian blue and Col2A1staining as well as Raman spectroscopy analysis. We observed through micro-CT scanning, Col2A1 staining, and histological analyses that delivery of BMP2 with coacervate could achieve a similar articular cartilage repair to that mediated by hMDSC-LBMP2/GFP. We also found that the addition of soluble fms-like tyrosine kinase-1 (sFLT-1) protein further improved the regenerative potential of hMDSCs/BMP2 delivered through the coacervate sustain release technology. Donor cells did not primarily contribute to the repaired articular cartilage since most of the repair cells are host derived as indicated by GFP staining.

**Conclusions:**

We conclude that the delivery of hMDSCs and BMP2 with the coacervate technology can achieve a similar cartilage repair relative to lenti-BMP2/GFP-mediated gene therapy. The use of coacervate technology to deliver BMP2/sFLT1 with hMDSCs for cartilage repair holds promise for possible clinical translation into an effective treatment modality for osteoarthritis and traumatic cartilage injury.

## Background

Osteoarthritis is a degenerative joint disease caused by loss of articular cartilage which affects 1/3 of the population aged 65 and older. It severely affects the daily activities of the patient. In the process of seeking a cure for this degenerative disease, clinicians and researcher have explored many different approaches for treatment of osteoarthritis. Using stem cell and growth factor gene therapy to regenerate articular cartilage has been one important field to develop approaches for treatment of osteoarthritis. It has been shown that bone marrow mesenchymal stem cell (BMMSCs) can repair articular cartilage when combined with certain scaffold materials and growth factors, such as bone morphogenetic protein 2 and 4 (BMP2,BMP4), Indian Hedgehog, and the transcription factor SOX-9 [1, 2]. The beneficial effects of BMMSCs have been shown to be not only related to their ability for direct differentiation to the chondrogenic lineage, but also to their immunosuppressive effects [3]. However, beneficial effects have not always been achieved, as was shown in a large animal (horse) model for which adding BMMSCs did not demonstrate better cartilage repair [4]. Adipose-derived stem cells have also been studied for cartilage repair when delivered with growth factors for gene therapy using different vectors or scaffolds [5–9]. A systematic review by Goldberg et al. demonstrated the advances of stem cells and cartilage repair [10]. Despite progress made towards the repair of cartilage, clinical application of these new treatments is still very limited. Muscle-derived stem cells (MDSCs) represent one kind of stem cell that has great potential for tissue engineering for musculoskeletal regeneration. Genetically modified murine MDSCs have been shown to effectively repair articular cartilage both in a monoiodoacetate (MIA)-induced osteoarthritis model and osteochondral defect model [11]. Also, blocking angiogenesis with soluble fms-like tyrosine kinase-1 (sFLT1)-transduced murine MDSCs has been shown to further enhance the cartilage repair capacity of bone morphogenetic protein 4 (BMP4)-transduced MDSCs [12, 13]. However, mMDSCs required gene modification to effectively undergo chondrogenic differentiation and promote articular cartilage repair. Human muscle-derived stem cells (hMDSCs) have been shown to be able to differentiate into chondrogenic, osteogenic, adipogenic, and myogenic lineages in vitro and have been able to enhance bone regeneration in vivo, when transduced with lentivirus expressing BMP2 [14]. It has also been shown that BMP2-transduced hMDSCs can repair critical size bone defects as efficiently as human BMMSCs [15]. HMDSCs’ bone regeneration capacities do not decline with aging [16]. However, whether hMDSCs could repair articular cartilage in vivo is still unknown. Coacervate is a slow release material formed by mixing solutions of heparin and a polycation, polyethylene argininylaspartate diglyceride (PEAD). It has been used for the delivery of fibroblast growth factor 2 (FGF2) to enhance angiogenesis in vivo successfully [17]. Coacervation is a self-assembly process driven by electrostatic forces that in this case allows near quantitative capturing of heparin-binding growth factors into the coacervate phase. Subsequently, it has been used to deliver different growth factors for cardiac infarction repair and effective wound healing [18–24]. It also has been utilized to sustained release of BMP2 for promoting murine MDSC-mediated ectopic bone formation [25]. In the current study, we compared gene therapy and a coacervate sustained delivery system to deliver BMP2 to test whether using coacervate-delivered BMP2 could enhance hMDSC-mediated cartilage repair using an MIA-induced osteoarthritis model, and the results were compared to lenti-BMP2/GFP-transduced hMDSC-mediated gene therapy.

## Materials and methods

The use of human tissue was approved by the IRB at the University of Texas Health Science Center at Houston (UTHealth), and all animal experiments and procedures were approved by the IACUC at UTHealth (AWC-15-0072).

### Cell isolation

Six populations of hMDSCs were isolated via a modified preplate technique, as previously described [26], from skeletal muscle biopsies purchased from the National Disease Research Interchange (NDRI). This pre-plate technique we used for isolation of MDSCs was modified from previously reported protocol [27–29]. Briefly, the muscle tissues purchased from NDRI were rinsed with Hanks Balanced Salt Solution (HBSS), and the fat and connective tissues were removed. Muscle tissues were cut into small pieces with scissors into slurry (1 mm in size) in HBSS solution. Then, the tissue slurry was centrifuged to remove HBSS, and pellets were weighed, and 1 ml of Collagenase XI was used for 0.1 g muscle. The tissues were digested with collagenase XI (0.2% weight/volume) (Sigma-Aldrich, Cat. #C7657), dispase (2.4 U/ml) (Invitrogen, Cat. #17105-041), and Trypsin-EDTA (0.1% weight/volume, Invitrogen, Cat. #15400-054) at 37 °C. The digestion time was 1 h for collagenase XI, 45 min for dispase, and 30 min for Trypsin-EDTA. The enzyme was removed by centrifugation at 2630 g for 5 min for each step. We added the dispase digestion step in our protocol compared to previous protocol. The cells were seeded in collagen I coated flasks (Sigma-Aldrich, Cat. #C9791) and incubated at 5% CO_2_ and 37 °C for 2 h. The un-adhered cells were transferred to another new collagen-coated flask. This step was repeated 5 more times until we obtained the preplate 6 (pp6). The pp6 cells eventually adhered and grew and were called hMDSCs [26]. The 6 populations of hMDSCs included three young populations (31-year-old female, hMDSC1; 21-year-old male, hMDSC2; 23-year-old male, hMDSC3) and three old populations (76-year-old female, hMDSC4; 78-year-old male, hMDSC5; 80-year-old male, hMDSC6) respectively. The hMDSCs were grown and maintained in proliferation medium (PM) that contained high glucose DMEM (Invitrogen) supplemented with 20% fetal bovine serum (FBS), 1% chicken embryo extract (CEE), and 1% penicillin/streptomycin.

### Construction of the lenti-viral-BMP2/GFP vector

A lenti-viral vector encoding for human BMP2 gene under the control of the human cytomegalovirus (CMV) promoter with a green fluorescent protein (GFP) tag, which was separated by an internal ribosome entry site (IRES) from the target gene, was constructed in collaboration with Dr. Bing Wang’s Laboratory. The GFP tag allowed us to monitor transduction efficiency and use fluorescence-activated cell sorting (FACS) to select transduced cells. Lenti-GFP (LGFP) and lenti-BMP2/GFP (LBMP2/GFP) viral vectors were packaged using 293 T cells (ATCC) using established protocol.

### Cell transduction

Human MDSCs were transduced with LBMP2/GFP or LGFP virus in the presence of polybrene (8 μg/ml) using 1:4 and 1:12 dilution with proliferation medium respectively at passage 8–10 for 16 h. At 24 h after transduction, transduction efficiency was observed under a fluorescent microscope. The transduction efficiency was about 50–60%. Cells were passaged 2 times after transduction and then subjected to GFP cell sorting (FACS) based on GFP fluorescence. After cell sorting, the cells were expanded in proliferation media. Supernatants were collected from different passages of each population and BMP2 secretion levels were measured using an ELISA (DBP200, R&D system). One population of young female cells (hMDSC1) was transduced with both LGFP and LBMP2/GFP for in vivo studies.

### In vitro chondrogenesis

In vitro chondrogenesis assay was performed for non-transduced or LBMP2/GFP-transduced hMDSCs using a 3D pellet culture method, as previously described [30]. Briefly, 6 populations of non-transduced cells and 6 populations of LBMP2/GFP-transduced cells were cultured in proliferation medium and expanded. 1.25 × 10^5^ cells from each population were aliquoted to 15 ml tubes in 4 replicates/population. The cells were centrifuged at 800*g* for 5 min and resuspended with complete chondrogenic medium (StemPro® Chondrogenesis Differentiation Kit, A1007101, ThermoFisher Scientific). The cells were then centrifuged at 500*g* for 5 min, and the lids of tubes were loosened ¼ turn to allow for oxygen exchange. Using this method, cells usually form pellets around 3 days of culture. Chondrogenic medium was changed every 2–3 days for 24 days. The pellets were fixed with neutral buffered formalin (NBF), rinsed once with PBS, then embedded in NEG freezing medium, snap frozen in liquid nitrogen, and stored at − 80 °C until sectioning, at which time 8-μm cryosections were cut. Pellets’ cultures were repeated three times for each population. Alcian blue staining was performed using online protocol (http://www.ihcworld.com/_protocols/special_stains/alcian_blue.htm.). Images were captured using a NIKON Cil microscope, and blue matrix was quantified using the NIKON NIS Element software. Collagen type II alpha 1 (Col2A1) immunohistochemistry (IHC) was also performed using goat anti-Col2A1 (SC7764, 1:50, Santa Cruz Biotechnology). In addition, Raman spectroscopy was utilized to quantitate sulfated cartilage matrix (proteoglycan aggrecan) at the Raman band ~ 1060 cm^−1^ (sulfate) and collagen at Raman band ~ 856 cm^−1^ (Proline) for the chondrogenic pellets derived from hMDSC5 and hMDSC6 before fixation by Dr. Xiaohong Bi’s laboratory.

### Preparation of coacervate and binding of BMP2 and sFLT1

Preparation of coacervate and binding of BMP2 and sFLT1 was carried out as follows. BMP2 (120-02C, PeproTech) and sFLT1 (ab54346, Abcam) were purchased and resuspended according to the manufacturer’s protocol to a concentration of 100 ng/μl in phosphate-buffered saline (PBS). Coacervate was formed using heparin and PEAD, which was synthesized by Dr. Yadong Wang’s lab, as previously described [31, 32]. We engineered our controlled delivery system to be more stable than the typical coacervate after a selection process that paired the polycation, PEAD, with heparin. We have previously demonstrated that the coacervate was present after 4 weeks in an infarcted myocardium [33]. BMP2 (500 ng) alone, or BMP2 plus sFLT1 (500 ng each), was first mixed with 12.5 μl of heparin (2 mg/ml) to allow proteins to bind to heparin, followed by addition of 12.5 μl of PEAD (10 mg/ml), and the complex became turbid which indicated the formation of coacervate. Then, each coacervate mixture was ready for combining with the different cell populations, as described below.

### In vivo articular cartilage (AC) repair using MIA-induced osteoarthritis model

In vivo cartilage repair was investigated using an MIA-induced global osteoarthritis model with delivery of BMP2 and hMDSCs using coacervate, and this delivery method was compared with LBMP2/GFP gene therapy. Thirty male nude rats (Taconic) of 12 weeks old were injected with 0.3 mg/150 g body weight MIA in 50 μl volume in the right knee (injured) according to our published paper [12], and the left knee (uninjured) was used as the normal control per our animal protocol approved by the IACUC at UTHealth. Two weeks after MIA injection, the rats were divided into 5 groups and injected in the injured knee joint with different combinations of cells/proteins or complexes (as stated below). Nude rats were divided into the following 5 groups (*N* = 6 each group).

*PBS + coacervate group*: PEAD (12.5 μl) was added to heparin (12.5 μl), and then PBS (25 μl) was added, followed by mixing and injection.

*BMP2 + PBS + hMDSC-LGFP (1 × 10*^*6*^*) group*: BMP2 (500 ng in 5 μl) was added to PBS (25 μl) and then added to the hMDSC-LGFP cell suspension (1 × 10^6^ cells in 20 μl PBS), followed by mixing prior to injection.

*PBS + hMDSC-LBMP2/GFP (1 × 10*^*6*^*) group*: PBS (25 μl) was added to 25 μl of cell suspension, for a total of 50 μl, and mixed prior to injection.

*BMP2 + coacervate + hMDSC-LGFP (1 × 10*^*6*^*) group*: BMP2 (500 ng in 5 μl) was mixed with heparin (12.5 μl), then PEAD was added (12.5 μl), and the complex was mixed with hMDSCs (1 × 10^6^) in 20 μl PBS before injection.

*BMP2 + sFLT1 + coacervate + hMDSC-LGFP group*: BMP2 (500 ng in 5 μl) and sFLT1 (500 ng in 5 μl) were mixed with heparin (12.5 μl) and then PEAD (12.5 μl) was added, and then the protein-loaded complex was mixed with the hMDSC-LGFP cell suspension (1 × 10^6^ in 15 μl PBS) just prior to knee joint injection. The nude rats were sacrificed at 12 weeks post-knee joint cell injection, and both the injured and uninjured knees were harvested and then fixed in NBF for 3 days for subsequent microCT and histology.

### MicroCT scanning and analysis

After fixation, both injured and uninjured knees were scanned using microCT (Viva CT 40, Scanco Medical) without contrast using 70 kvp, 112 μA, and a 30-μm voxel size. 3D images of the whole knee joint were reconstructed using Gauss = 0.8, Sigma = 1, and a threshold of 200 using the same dimensions for subsequent analysis. After 3D reconstruction, the empty gap of each knee joint of 3D image was measured using image J software. The bigger gap indicated more severe the cartilage erosion. The knee joint gap differences were calculated using injured knee gap subtracted non-injured knee of the same rat and compared between groups.

### Histology

After microCT, the knee joints were decalcified using 10% ethylenediaminetetraacetic acid disodium (EDTA) plus 1% sodium hydroxide for 3 months. Whole knee joints were cut in the middle sagittally, dehydrated, and then paraffin embedded so that the middle of the joint (groove level) and the edge of the joint (condyle level) could be viewed. Both levels of sections were used for quantification and histology score. Paraffin sections of 5 μm were cut. H&E staining and Alcian blue staining were performed per the following IHC WORLD protocols respectively: http://www.ihcworld.com/_protocols/special_stains/h&e_ellis.htm

http://www.ihcworld.com/_protocols/special_stains/alcian_blue.htm.

Toluidine blue staining was performed using IHC world protocol:

http://www.ihcworld.com/_protocols/special_stains/toluidine_blue.htm

Images were captured for entire area of cartilage surface of each animal at both middle (groove) and edge (condyle) at × 200 magnification. The summed area of each pathological change was measured with Image J for scoring. Histology score was given using Osteoarthritis Research Society International (OARSI) grading (1–6) and staging (1–4) criteria [34]. The grading and staging were performed blindly. If the score is higher, it indicates worse cartilage repair.

### Immunohistochemistry

IHC staining of GFP-positive cells was used to reveal donor cells in the regenerated cartilage and Col2 staining was used to detect specific cartilage matrix collagen 2. Briefly, after deparaffinization, washing, and blocking with 5% donkey serum in PBS, sections were incubated with rabbit anti-GFP antibody (ab290, Abcam, 1:1000 dilution) and rabbit anti-Col2 (ab34712, Abcam: 1:400 dilution) in 5% donkey serum overnight. For Col2 staining, antigen retrieval was performed using 2% hyaluronidase (H3506-5G, Sigma,**)** in PBS at room temperature for 30 min, followed by washing with PBS three times before blocking and incubation with primary antibody. The following day, sections were treated with 0.5% H_2_O_2_ in PBS for 30 min at room temperature, washed in PBS, and then incubated with goat anti-rabbit biotin (BA 1000, Vector Laboratories, Burlingame, CA, USA, 1:200 dilution) for 2 h at room temperature. After three washes, each slide was incubated with ABC reagent (PK 7200, Elite ABC kits, Vector Laboratories) for 2 h at room temperature. After three washes with PBS, diaminobenzidine (DAB) staining (SK-4100, Vector Laboratories) was used to visualize the GFP-positive cells. Hematoxylin (H-3404, Vector laboratories) counterstaining was performed following the DAB color reaction.

### Statistical analysis

One-way analysis of variance (ANOVA) followed by Tukey’s post hoc multiple test was used to analyze multiple quantitative data using GraphPad Prism 7. Two-side Student *T* test was used to compare the two groups. Wilcoxon rank-sum non-parametric test was used for microCT and histology score analysis due to high deviation of the parameters. In brief, the two comparison group values were ranked from small to large, and the numerical rank sum of each group was compared to the Wilcoxon rank-sum table; the *P* value was determined based on the upper tail and lower tails of the comparison groups. If the sum of one group was larger than the upper tail value, then we deemed *P* < 0.05 or *P* < 0.01 to be statistically different. Similarly, if the sum of one group was lower than lower tail probability value, we also deemed *P* < 0.05 or *P* < 0.01 was statistically different. Overall, a value of *P* < 0.05 was considered statistically significant for all statistical analysis method.

## Results

### Lenti-viral transduction efficiency of hMDSCs

**Fig. 1:**
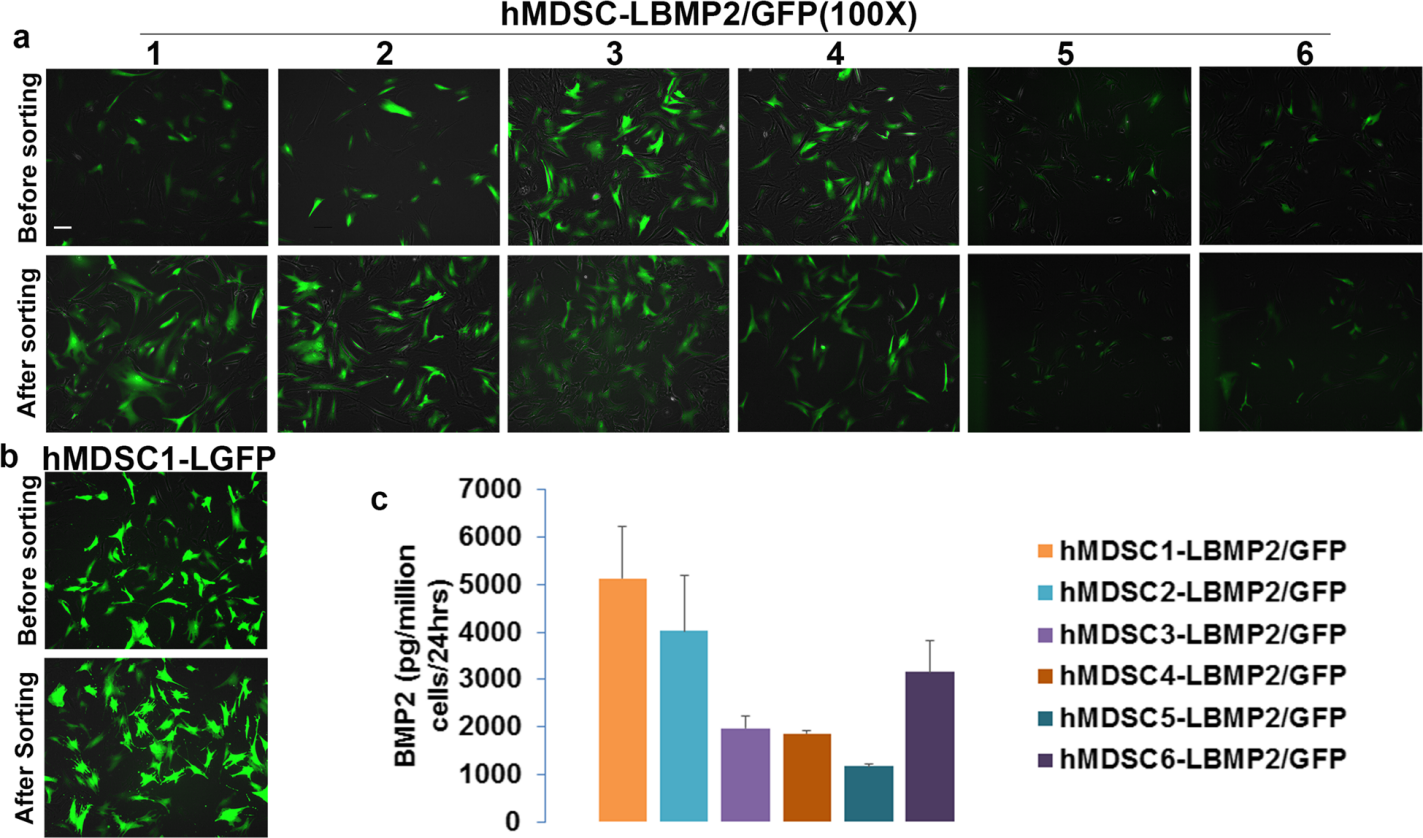
Lenti-viral transduction and flow cytometry sorting of GFP-positive cells of lenti-BMP2/GFP-transduced hMDSCs and lenti-GFP-transduced hMDSCs**. a** GFP fluorescent images of 6 populations of lenti-BMP2/GFP transduced cells before and after cells sorting. **b** GFP fluorescent images of lenti-GFP-transduced hMDSCs before and after cell sorting. **c.** BMP2 secretion level by ELISA of lenti-BMP2/GFP-transduced hMDSCs after GFP sorting. Scale bar = 100 μm

We found that lenti-BMP2/GFP transduction efficiency was about 50–60% for 6 populations of hMDSCs. The cells proliferated well at 1:4 dilution. After GFP flow cytometry cell sorting, the GFP-positive rate was increased to 90–95% after transduction; however, GFP fluorescent intensity became weaker after sorting (Fig. 1a). For the lenti-GFP transduction, the transduction efficiency was about 80% when we used 1:12 dilution. After GFP sorting, the GFP-positive rate increased to around 95% based on fluorescent imaging (Fig. 1b). The BMP2 secretion levels for the sorted lenti-BMP2/GFP-transduced cells range from 1171 to 5123 pg/million cells/24 h. The level of BMP2 secretion by the transduced was the highest with the hMDSC population 1 (hMDSC1) and the lowest with hMDSC population 5 (hMDSC5) (Fig. 1c).

### LBMP2/GFP-transduced hMDSCs exhibited higher chondrogenic differentiation capacity compared to non-transduced hMDSCs

**Fig. 2:**
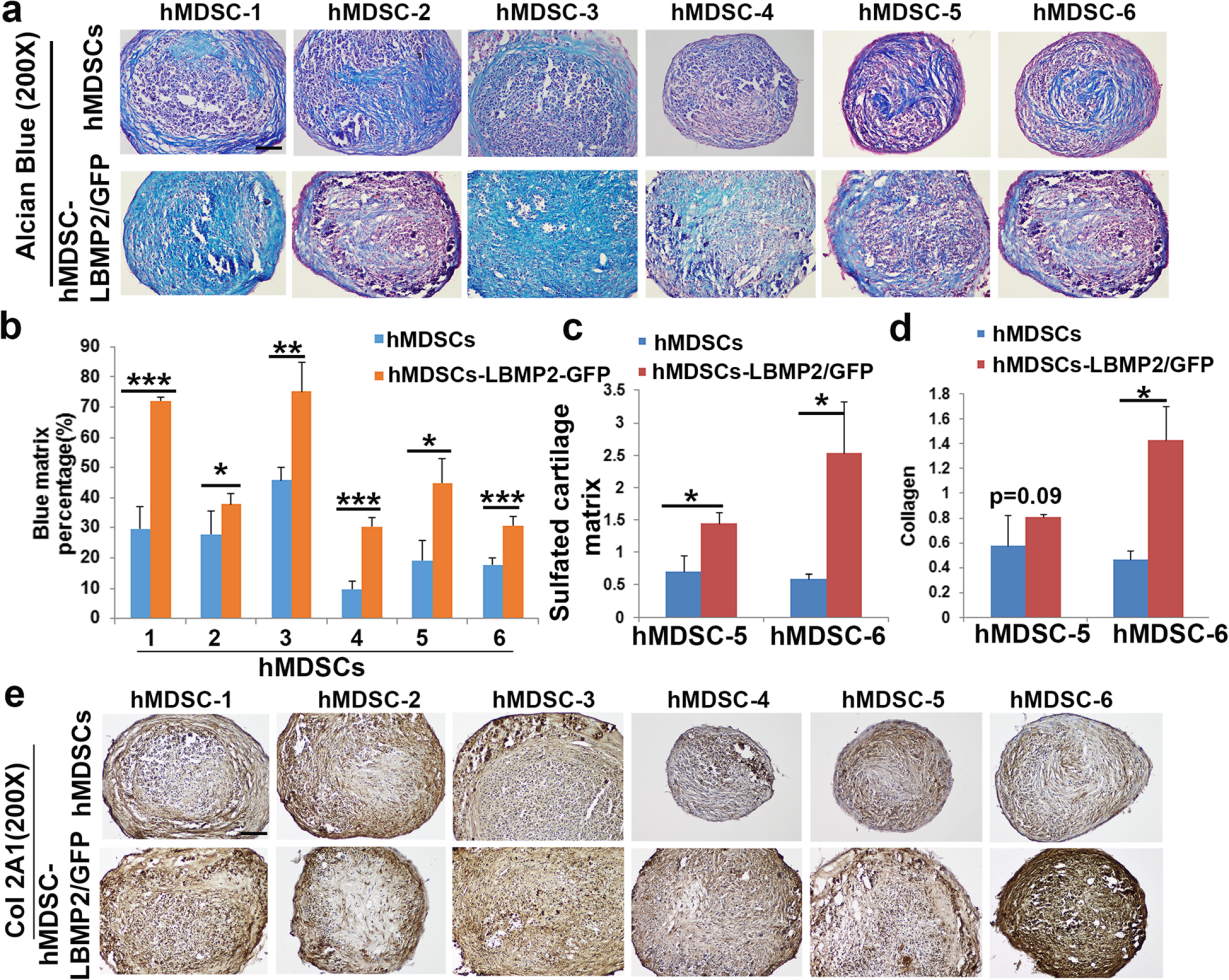
**a** Alcian blue staining. Cartilage matrix including acidic sulfated mucosubstances and hyaluronic acid are stained in blue. **b** Quantification of blue matrix showed significantly higher percentages of blue matrix in the LBMP2/GFP-transduced cells compared to non-transduced hMDSCs. Scale bar = 100 μm. **c** Raman spectroscopy quantification indicated significantly higher amount of sulfate cartilage matrix (proteoglycan aggrecan) in LBMP2/GFP-transduced hMDSCs than in non-transduced hMDSCs. **d** Raman spectroscopy indicated higher collagen content in the LBMP2/GFP-transduced hMDSCs, although differences were not always significant. **e** Collagen 2A1 (Col2A1) immunohistochemistry indicated stronger Col2A1 staining in the LBMP2/GFP-transduced hMDSCs compared to respective non-transduced hMDSCs. Scale bar = 100 μm. *P < 0.05, **P < 0.01, ***P < 0.001

We performed 3D pellet culture assays to test the chondrogenic potential of 6 populations of human cells before and after lenti-BMP2/GFP transduction. After 24 days of culturing in chondrogenic medium, Alcian blue staining showed more Alcian blue-positive chondrogenic matrix in the LBMP2/GFP-transduced hMDSC pellets compared to non-transduced hMDSC pellets in all cell populations tested (Fig. 2a). Quantification of the percentage of blue matrix demonstrated significantly higher chondrogenesis in the LBMP2/GFP-transduced relative to non-transduced cells for all 6 populations of hMDSCs (Fig. 2b). Raman spectroscopy measurement indicated the LBMP2/GFP-transduced hMDSCs contained the higher amount of sulfated cartilage matrix (proteoglycan aggrecan) and collagen when compared to non-transduced hMDSCs, although the differences were not always statistically significant (Fig. 2c, d). Col2A1 immunohistochemistry showed more Col2A1 in the LBMP2/GFP-transduced hMDSCs compared to non-transduced hMDSCs (Fig. 2e).

### MicroCT analyses revealed that cartilage erosion after MIA-induced injury was decreased after treatment with hMDSCs with BMP2

**Fig. 3:**
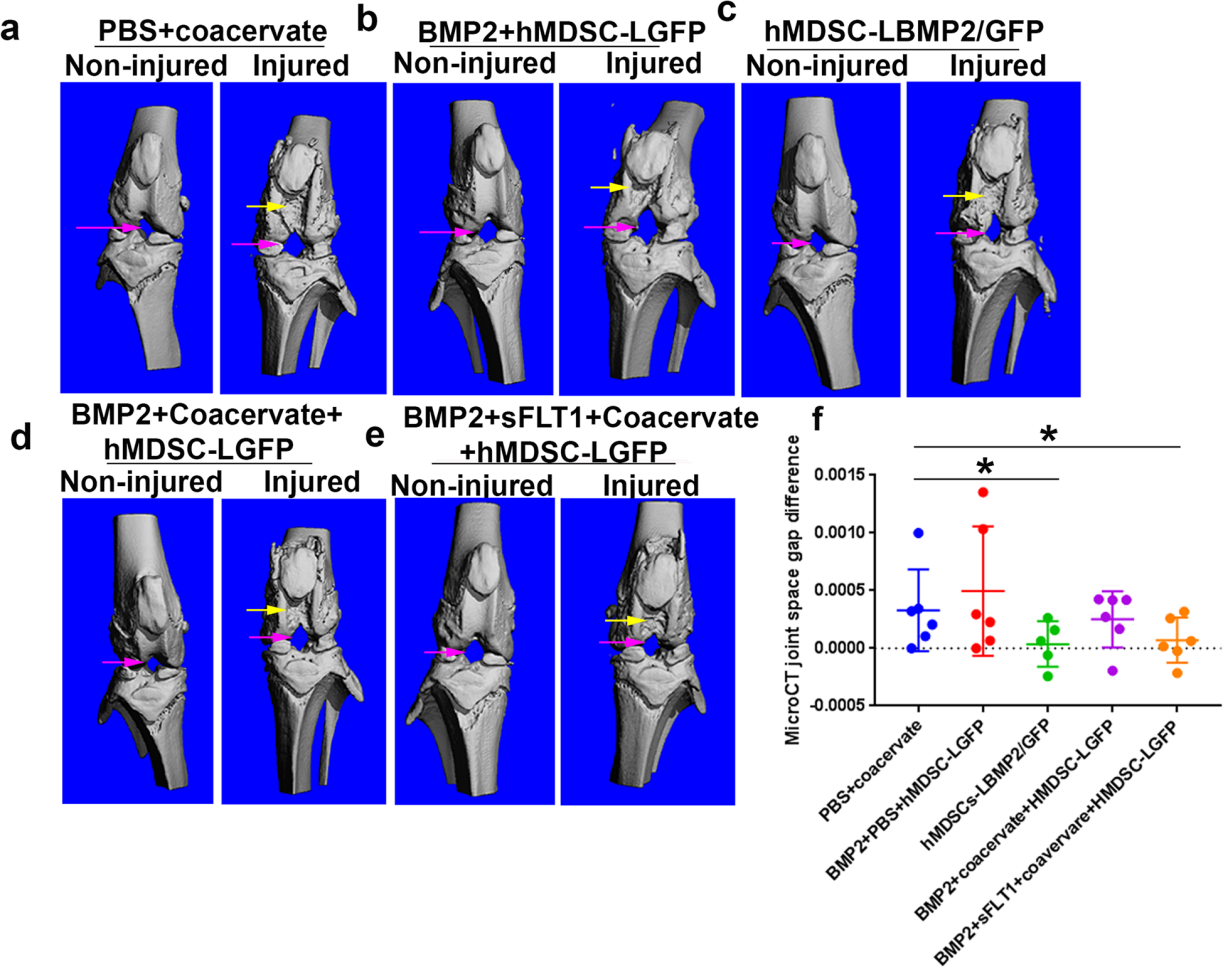
MicroCT 3D images and knee joint space quantification. **a**–**e** Representative MicroCT images of knee joints for non-injured and injured sides of each group. It is obvious that there is cartilage erosion in the injured side and that the femoral condyle and trochlear groove cartilage are exposed compared to that of the non-injured side for all 5 groups. Subchondral cancellous bone is visible in the injured side. All of the hMDSC-treated groups appeared to be smoother than the PBS group. Pink arrows pointed to joint empty space; yellow arrows pointed to cartilage erosion. **f** Quantification of knee joint space differences between non-injured and injured sides among groups. The joint space differences between non-injured knees and injured knees were significantly smaller in the hMDSC-LBMP2/GFP group and the BMP2 + sFLT1 + coacervate+ hMDSC-LGFP group when compared to the PBS group. No significant differences were found for the other groups. *P < 0.05, Wilcoxon rank-sum test

MicroCT 3D images showed that each of the non-injured knee joints had smooth joint surfaces. The trochlear groove was smooth. The injured knees had different extents of joint cartilage erosion, as the subchondral bones were obviously visible in the trochlear groove and femoral condyles and tibia plateau. Knee joint space changed in the injured side of the knees in all groups compared to the non-injured side of knee (Fig. 3a–e). Quantification of knee joint space differences (injured knee − non-injured knee) indicated that the hMDSC-LBMP2/GFP group and the BMP2 + sFLT1 + coacervate + hMDSC-LGFP group had significantly smaller joint space difference which is indicative of better healing compared to the PBS + coacervate group (Fig. 3f). No significant differences were found for the other groups.

### Histology grade scores were lower in all the hMDSC-injected groups

**Fig. 4:**
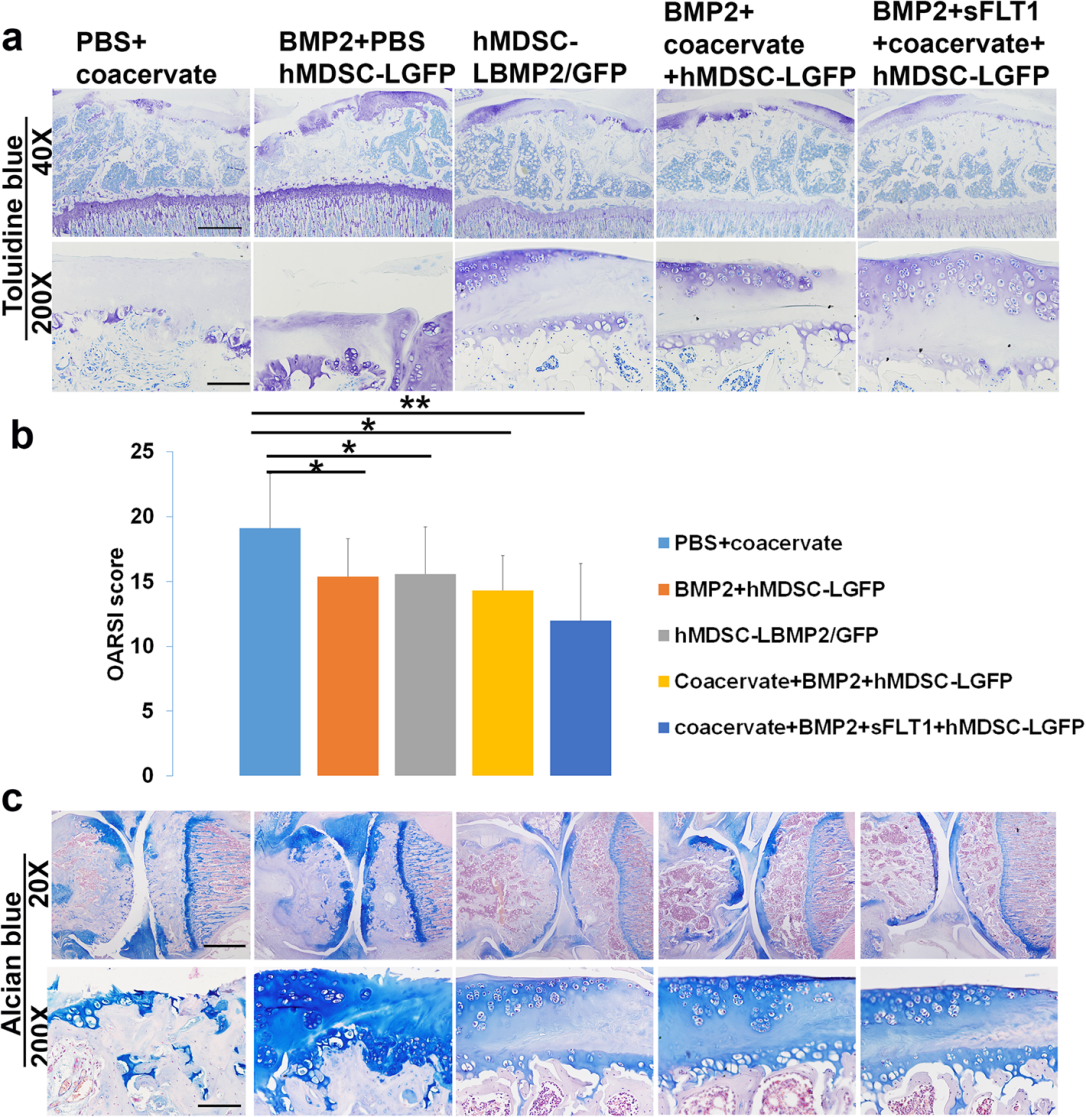
Evaluation of histology score using Toluidine blue and Alcian blue staining. **a** Toluidine blue staining of the 5 different groups at 3 months after cell (or PBS + coacervate) injection. Upper panel showed entire tibia plateau at the condyle axial level, scale bar = 500 μm. Lower panel showed high magnification, scale bar = 100 μm. The PBS + coacervate group showed that the knee joint cartilage layer was almost completely destroyed. Very limited chondrocytes were left at the joint cartilage. All other groups showed cartilage regeneration at cartilage surface to different extent. **b** OARSI histology scores based on Toluidine blue staining in each of the 5 groups. All groups that were injected with hMDSCs had lower scores compared to the PBS + coacervate control group. *P < 0.05, **P < 0.01. Wilcoxon rank-sum test. **c** Alcian blue staining showed similar results as Toluidine blue staining. Upper panel showed the morphology of cartilage of entire knee at condyle axial level. Scale bar = 1000 μm. Lower panel showed chondrocytes morphology at the articular surface of each group, scale bar = 100 μm

We performed Toluidine blue and Alcian blue staining and histology scoring using the OARSI grading system [34] to measure the histology grade of the knee joint cartilage, including the distal femur and proximal tibia articular cartilage. This system included 6 histological grades and 4 histological stages. The total score (grade multiplied by stage) ranged from 1 point (normal articular cartilage) to 24 points (no repair). The results indicated that all hMDSC-injected groups showed significant cartilage repair relative to the PBS + coacervate group that had no cells injected. There were minimal chondrocytes on the cartilage surface of the PBS + coacervate group from both edges of articular cartilage. In the PBS + BMP2 + hMDSC-LGFP group, we found chondrocytes clustered on the cartilage surface from both edges of the cartilage surface. In the hMDSC-LBMP2/GFP group, we found more Toluidine blue-positive chondrocytes at the cartilage layer. In the BMP2 + coacervate + hMDSC-LGFP group, we observed more regenerated chondrocytes on the cartilage surface. Finally, in the BMP2 + sFLT1 + coacervate + hMDSC-LGFP group, we observed more newly generated chondrocytes at the cartilage surface than in the PBS + coacervate group (Fig. 4a). However, none of the groups showed completely cartilage regeneration on the cartilage surface. Quantification of the histology scores showed that all the hMDSC-treated groups demonstrated significantly improved histology scores (lower value) compared to the PBS + coacervate group. Coacervate-delivered BMP2 plus hMDSCs achieved slightly better scores than the hMDSC-LBMP2/GFP group but showed no statistical differences. The BMP2 + sFLT1 + coacervate + hMDSC-LGFP group yielded the best histology scores (Fig. 4b). Alcian blue staining demonstrated similar results as Toluidine blue staining (Fig. 4c).

### Cartilage repair was improved in hMDSC-LBMP2/GFP and coacervate-delivered BMP2 and sFlt1 in combination with hMDSCs groups

**Fig. 5:**
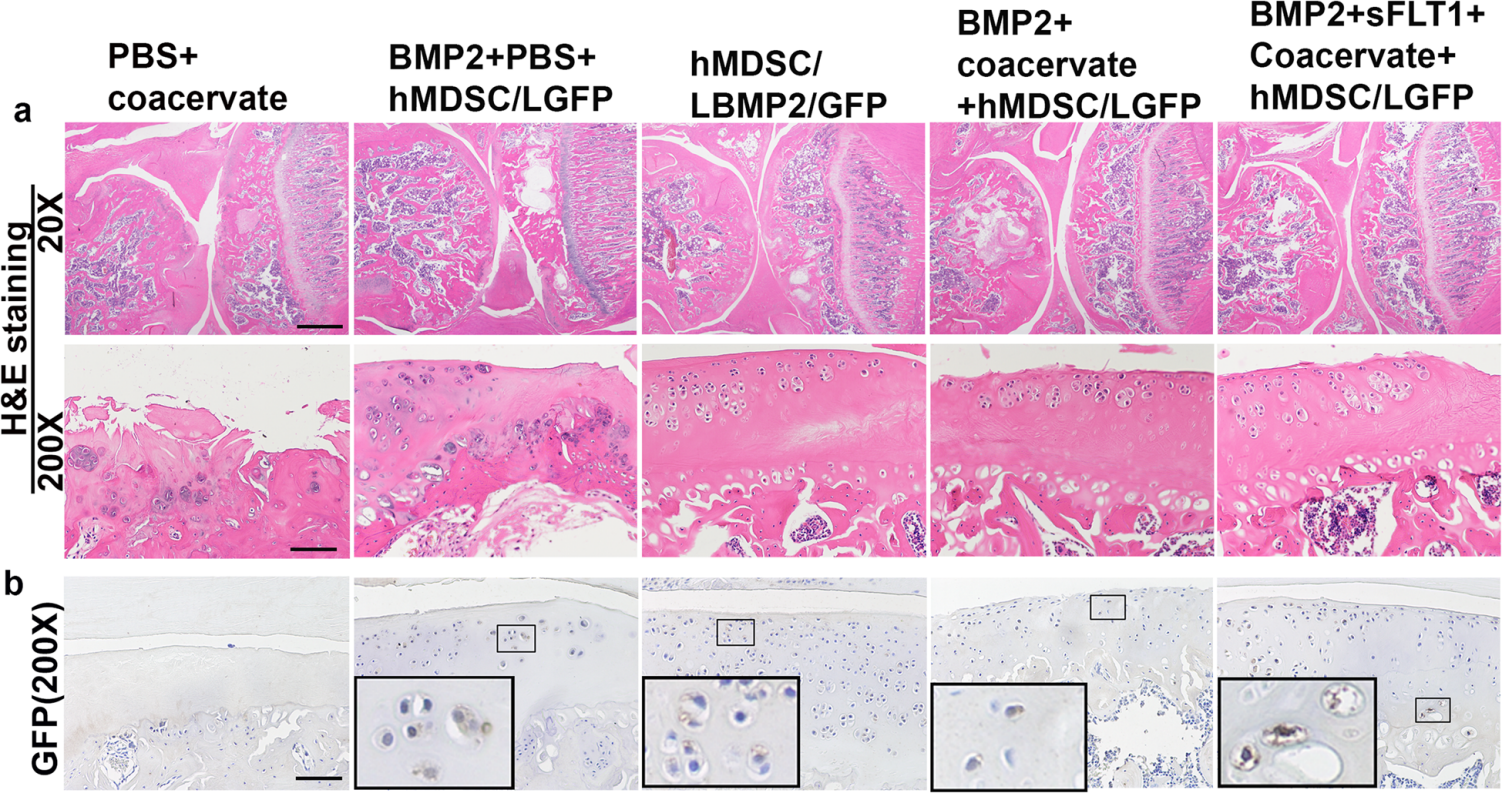
H&E and GFP staining. **a** H&E staining indicated an almost absence of chondrocytes in the cartilage layer in the PBS + coacervate group. All the other groups showed some chondrocytes in the injured cartilage. Scale bar in upper panel = 1000 μm, lower panel = 100 μm. **b** GFP staining indicated that few GFP-positive cells were found in the regenerated cartilage. Insets are the enlarged boxed area to show GFP-positive cells. The majority of the cells are from the host and are GFP-negative. Scale bar = 100 μm

We performed H&E staining to look at the general articular cartilage structure for each group. We found that the PBS + coacervate control group had very few chondrocytes in the cartilage area from both edges of articular cartilage, which implied that the majority of the cartilage cells had died. Subchondral bone was exposed in some areas. In the BMP2 + hMDSC-LGFP group, we found regenerated chondrocytes in clusters. Regenerated AC with chondrocytes was observed, but there was still much of the joint surface without cells. In the hMDSC-LBMP2/GFP group, we found more AC areas with regenerated cartilage cells. In the BMP2 + coacervate + hMDSC-LGFP group, we found similar articular cartilage regeneration compared to the hMDSC-LBMP2/GFP group. There were more cells in the superficial zone and fewer cells in the middle zone. In the BMP2 + sFLT1 + coacervate + hMDSC-LGFP group, we found better articular cartilage structure (Fig. 5a). Of note, we did not find residual coacervate in the knee cartilage after 12 weeks post-injection, which indicated that the coacervate was absorbed.

### Donor cells contributed to the regenerated cartilage, but host cells contributed the majority of regenerated chondrocytes

In order to trace whether the transplanted hMDSCs differentiated into chondrocytes and contributed to the regenerated cartilage, we performed GFP immunohistochemistry. We found scattered GFP-positive cells in the regenerated cartilage area on the articular surface in all the groups that had hMDSCs injected. However, GFP-positive cells were relatively fewer in number compared to GFP-negative cells indicating that the regenerated cartilage is composed primarily of host-derived chondrocytes (Fig. 5b). Insets are the enlarged boxed area from the original image showing the GFP+ cells.

### Col2 staining demonstrated the regeneration of chondrocytes in the regenerated cartilage surface

**Fig. 6:**
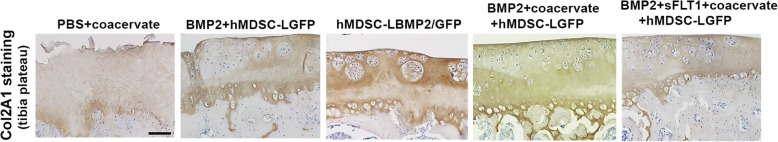
Col2 staining. Brown color showed Col2-positive cartilage matrix. We observed very few chondrocytes in the PBS + coacervate group, and the residual matrix was Col2A1-positive. The BMP2 + hMDSC-LGFP group showed chondrocyte regeneration, although only partial, and Col2A1 staining was positive in certain areas. In the hMDSC-LBMP2/GFP group, Col2-positive chondrocytes were found in cluster. In the BMP2 + coacervate + hMDSC-LGFP group, Col2-positive chondrocytes distributed mainly on the superfacial zone and middle zone, zones near tide marker showed no chondrocytes. The BMP2 + sFLT1 + hMDSC-LGFP group showed slightly better chondrocytes distribution than the hMDSC-LBMP2/GFP group and BMP2 + coacervate+ hMDSC-LGFP group. Scale bar = 100 μm

In order to test whether the newly generated cartilage on AC surface has cartilage properties, we performed Col2 staining, which labels typical cartilage matrix. We observed few chondrocytes at the edge of articular cartilage in the PBS + coacervate group that are Col2-positive, and the residual matrix was less intense Col 2-positive. In the BMP2 + hMDSC-LGFP group, we found chondrocyte regeneration, although only partial, and Col2 staining was strongly positive in certain areas. In the hMDSC-LBMP2/GFP group, we found more chondrocytes that were Col2-positive on the articular cartilage surface. In the BMP2 + coacervate + hMDSC-LGFP group, we also observed extensive expression of Col2 in the regenerated cartilage. Many Col2-positive chondrocytes were identified. In the BMP2 + sFLT1 + hMDSC-LGFP group, there were more Col2-positive chondrocytes compared to other groups (Fig. 6).

## Discussion

In this study, we investigated chondrogenic potential of 6 human populations of hMDSCs and found that the LBMP2-transduced hMDSCs exhibited enhanced chondrogenic differentiation in vitro compared to the non-transduced cells. Furthermore, we tested the cartilage repair capacity of hMDSC-LGFP plus free BMP2 and hMDSC-LGFP combined with coacervate-delivered BMP2, and compared the results with hMDSC-LBMP2/GFP group to test their cartilage repair capacity in vivo. We also tested dual delivery of BMP2 and sFLT1 with the coacervate for cartilage repair. Our results indicated that all the hMDSC injection groups demonstrated partial cartilage repair, although to different extents. The hMDSC-LBMP2/GFP group showed both improvement in cartilage erosion (revealed by microCT 3D images of joint gaps and histological score) relative to the PBS + coacervate group. Coacervate-delivered BMP2 plus hMDSC-LGFP achieved similar histology scores compared to the hMDSC-LBMP2/GFP gene therapy approach. Coacervate dual delivery of BMP2 and sFLT1 plus hMDSC-LGFP achieved the best AC regeneration observed by microCT and histology scores. Many different stem cells have been used in the treatment of arthritis experimentally or clinical trial. Bone marrow aspirate concentrate embedded in hyaluronic acid scaffold has been used for cartilage injury in clinic and proved to be good to excellent outcome at long-term follow-up for small to large cartilage lesions, and single to multiple lesions [35]. Mesenchymal stem cells loaded on Tantalum have been shown to improve cartilage repair in a large osteochondral defect model in goat [36]. Adipose stem cells in combination with different scaffold have been shown to ameliorate cartilage regeneration in a different animal models [37–39]. Adipose stem cells have been tested in phase II clinical trial for knee osteoarthritis treatment, and the results have indicated the improvement of joint function, pain, quality of life, and cartilage regeneration [40]. Adipose stem cells have also been shown beneficial effect for microfracture-mediated cartilage repair in clinical patient [41]. Previously, we have shown that murine MDSCs can efficiently repair rat MIA-induced osteoarthritis [12, 42] when transduced with retroviral-BMP4/GFP. Our current study presents findings for the first time the use of human MDSCs to repair cartilage. In this study, we first tested whether hMDSC-BMP2/GFP has enhanced chondrogenic differentiation ability. We tested 6 populations of hMDSCs and found that LBMP2/GFP-transduced cells all demonstrated enhanced in vitro chondrogenesis compared to non-transduced cells, as indicated by Alcian blue and Col2A staining as well as Raman spectrometry quantification of sulfated cartilage matrix and collagen. These results encouraged us to perform in vivo experiments. Furthermore, we found that all groups, which had hMDSC treatments, showed improved AC repair compared to the PBS control group. We found that the hMDSC-LBMP2/GFP group had better cartilage repair than the PBS + coacervate group, which demonstrated limited cartilage repair after MIA-induced osteoarthritis. However, the direct contribution of the hMDSCs to the repaired cartilage was limited, as only a few GFP-positive cells were found in the repaired cartilage. Several reasons may explain the lower GFP detection rate on the repaired cartilage. One possibility is that the GFP from the transduced hMDSCs is being gradually lost during cell division. It is also possible because the GFP-positive cell percentage is not 100% even after cell sorting. The loss of GFP expression may also be attributed to the long period of decalcification, since for this study we have decalcified for 3 months using a 10% EDTA plus 1% NaOH solution. We only decalcified for 1 month for regenerated bone tissues mediated by murine MDSC/BMP4/GFP [15, 30]. However, we have ruled out the possibility of the GFP antibody as we have used this antibody for murine MDSCs /BMP4GFP transplantation and have shown robust GFP-positive staining [15, 30]. The hMDSCs mainly served as a BMP2 delivery vehicle. Donor cell contribution to cartilage repair has been shown to vary by the use of different stem cells. It has been shown that murine MDSCs transduced with BMP4/GFP can directly differentiate into chondrocytes in MIA-induced osteoarthritis [12] and osteochondral defects [43], but also in a small percentage compared to those from host cells. Bone marrow mesenchymal stem cells injected in the knee joint in MIA-induced model reduced pain, but did not improve structural damage to the cartilage and subchondral bone and synovitis. Cell tracking indicated cell survival until 2 weeks [44]. While there has been concern with respect to gene therapy and biosafety, we, therefore, also tested whether using biomaterials to deliver BMP2 can also achieve similar cartilage repair effects of lenti-BMP2-transduced hMDSCs. We used coacervate, a polymer biomaterial that has been shown to successfully deliver other growth factors for tissue repair [32]. Indeed, we found that using coacervate to deliver 500 ng BMP2 in combination with hMDSC-LGFP (BMP2 + coacervate + hMDSC-LGFP group) achieved similar cartilage repair in terms of microCT analysis and histology scores as lenti-BMP2/GFP-transduced hMDSCs. The biomaterial is injectable and with no residue found in the injected joint 12 weeks after intra-articular injection. Furthermore, we took advantage of the biomaterial’s ability to deliver growth factors and performed dual delivery of BMP2 (500 ng) and sFLT1 (500 ng), as it is known that blocking angiogenesis can further enhance cartilage repair [12, 13]. Our results demonstrated that adding sFLT1 enhanced cartilage repair with hMDSCs and BMP2, as demonstrated by microCT and histology. These results support the feasibility of using hMDSCs and growth factor without the need of gene transfer for cartilage repair. Finally, we need to point out that none of the treatment regimen was able to completely repair entire damaged cartilage surface. The possible reason may be because the disease progression continues until 12 weeks after MIA-induced arthritis [45]. The other reason may be due to the fact when the cells were injected into the joint space, and only those cells that attached to the cartilage surface will participate in the cartilage repair. Other injected cells may only exert paracrine effect. Therefore, more researches are warranted to further enhance the cartilage repair mediated by hMDSCs in combination with growth factors such as optimization of dose of BMPs and cell numbers and improvement of cell survival and the use multiple injections.

## Conclusion

We found LBMP2/GFP-transduced hMDSCs have enhanced chondrogenic capacity compared to non-transduced hMDSCs in vitro. The delivery of BMP2 together with hMDSCs significantly improved cartilage repair. The delivery of BMP2 using both gene therapy and coacervate biomaterial enhanced hMDSC-mediated cartilage repair. Blocking angiogenesis by co-delivery of sFLT1 along with BMP2 plus hMDSC-LGFP achieved the best results for cartilage repair. Endogenous cells recruited by BMP2 contributed more to the cartilage repair than the transplanted cells. Our results support the application of hMDSCs for cartilage repair using both gene therapy and biomaterials to deliver growth factors.

## Data Availability

The datasets used or analyzed (or both) during the current study are available from the corresponding author on reasonable request.

## Abbreviations

MDSC: Muscle-derived stem cells
BMP2: Bone morphogenetic protein 2
GFP: Green fluorescent protein
MicroCT: Micro-computed tomography
Col2: Collagen 2
sFLT1: Soluble fms-like tyrosine kinase-1
PEAD: Polyethylene argininylaspartate diglyceride
MIA: Monoiodoacetate
IRES: Internal ribosomal entry site
FACS: Fluorescent activated cell sorting
PBS: Phosphate-buffered saline
ANOVA: Analysis of variance
DAB: Diaminobenzidine
NBF: Neutral buffered formalin
